# Supplementary material for: Wireless ear EEG to monitor drowsiness
Source: Nat Commun. 2024 Aug 2;15:6520. doi: 10.1038/s41467-024-48682-7 (PMC11297174; doi:10.1038/s41467-024-48682-7)
Supplement: Supplementary file 1 — Supplementary Information [file 41467_2024_48682_MOESM1_ESM.pdf]

Wireless Ear EEG to Monitor Drowsiness

Supplement

I. Supplemental Results

a. Earpiece Design

The earpiece design began with custom molded earpieces from five volunteers (three male and two female). Initial impedance and EEG measurements from these earpieces supplemented ear canal measurements from large populations [45] to highlight the best locations for low-impedance electrode-skin contact. From here, electrode sizes and locations on a user-generic earpiece design were iteratively optimized to maximize electrode area (thus minimizing electrode impedance) while minimizing channel to channel correlation. Furthermore, electrode locations were also selected to not interfere with the normal morphological ear-canal changes that occur with age. Small, medium, large earpieces were designed and fabricated to ensure comfortable, electrode contact across a large population. Small and large earpieces were generated by scaling key earpiece features (ear canal aperture, isthmus, and length) by the standard deviations reported in [1], [2]. The small in-ear and out-ear electrodes were 50 mm^2 and 2 cm^2, respectively. The large in-ear and out-ear electrodes were 65 mm^2 and 4 cm^2, respectively. Electrode impedances across earpiece sizes were roughly equivalent, implying the electrodes are not making full contact. This is an acceptable outcome, because it is likely that electrodes will contact different ear canals in different ways. Maximizing electrode surface area is a way to guarantee that at least some part of the electrode will make reasonable contact (<1 MΩ) regardless of the subject.

b. Electrode Impedance comparisons

Electrode impedance is unchanged in the presence of naturally occurring cerumen (ear wax and oil). To better understand how electrode behavior may change in the presence of cerumen in real human ears, electrode impedance was measured initially without any skin or earpiece cleaning and again with a simple ear and earpiece cleaning step (Fig. S1). Ears and electrodes were cleaned with isopropyl alcohol and kimwipes to remove any physical detritus. Final measurement results (N=8) indicated no meaningful difference between the 'uncleaned' and 'cleaned' scenarios. This is likely due to the large surface area, capacitive electrodes, which do not rely on a direct faradaic charge transfer.

The ear-skin impedance also does not noticeably degrade over time. Electrode impedance was measured the day they were completed and one week after (Fig. S2). When comparing measurements across the same earpiece and same subject (N=4), ESI indicated no meaningful difference as expected with gold plated electrodes. It is important to note that during the week between ESI measurements, the electrodes underwent heavy use in various ExG exploratory tasks and multiple drowsiness trials, repeatably exposing then to cerumen, cleaning solution, and various other skin detritus.

Dry electrode impedance is slightly larger than that of commercial gold cup electrodes (Fig. S3). At 50 Hz, the in-ear electrodes achieve an impedance of 109 kΩ whereas commercial wet electrodes (without skin abrasion) have an impedance of 31.7 kΩ. While this difference of impedance may not seriously affect measured signal amplitude, dry electrode interfaces are more susceptible to motion artifacts.

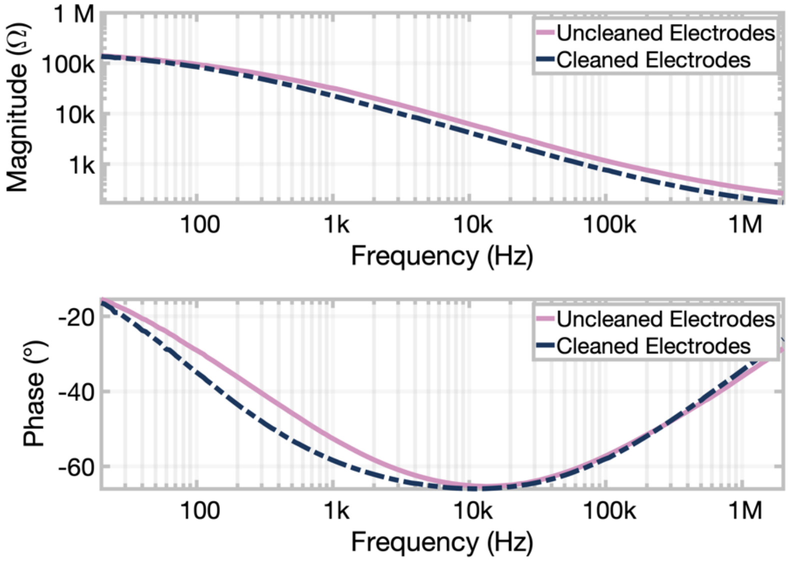

Figure S1: In ear electrode impedance magnitude and phase before and after skin/electrode cleaning (N = 8 for both measurements).

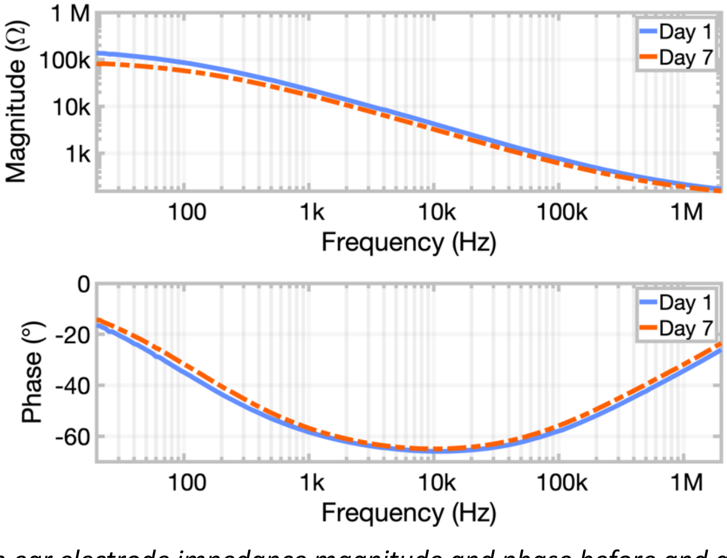

Figure S2: In ear electrode impedance magnitude and phase before and after a week of repeated use (N = 4 for both measurements). Measurements were conducted using the same earpiece and same subject.

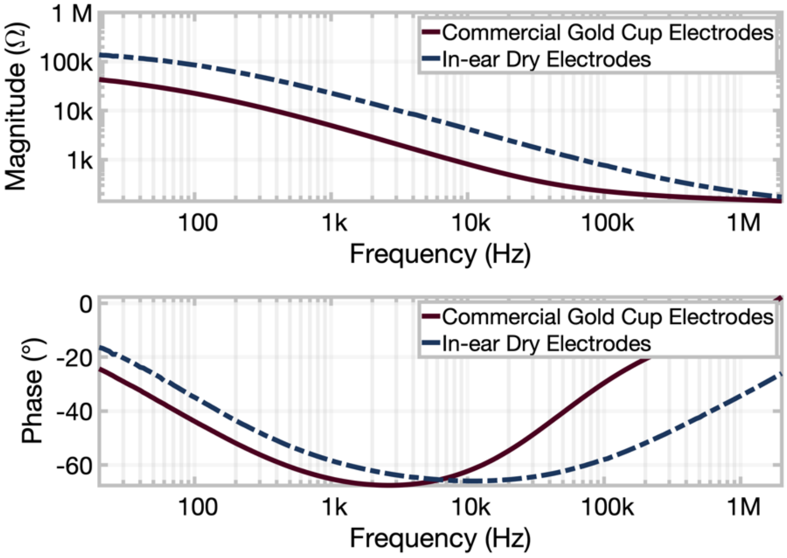

Figure S3: Commercial gold cup electrode impedance (with hydrogel) In ear electrode impedance (N=4).

c. Electrode Impedance Settling

In-ear dry electrodes tend to have settling times, but initial settling should be on the order of 2 minutes (based off the electrode model in Fig. 3e). Longer settling activity (on the order of 10's of minutes) is more likely due to sweat formation at the electrode-skin interface. Figure S4 plots In-ear electrode impedance at 50 Hz over the course of 34 minutes of wear. The subject was seated comfortably by the LCR meter (Keysight E4980), stood up and walked around in the middle of the measurement, and then sat back down to continue measuring long term electrode impedance. Initially, ESI at 50 Hz was ~610 kΩ and by the end of the measurement, ESI had dropped to ~510 kΩ. The measurement setup did not allow for simultaneous walking and impedance measurement so future work would involve monitoring impedance and EEG at the same time to track motion artifacts associated with impedance changes.

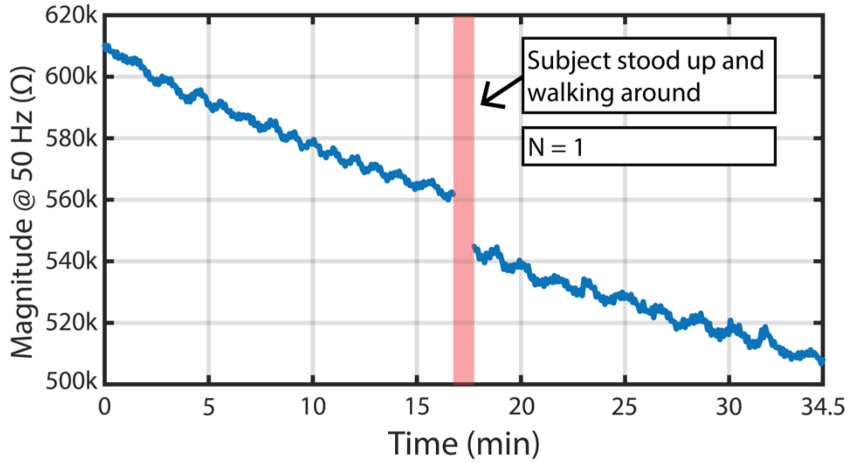

Figure S4: In-ear electrode impedance at 50 Hz over the course of 34 minutes of wear. The subject was seated for the first half of the measurement, stood up at the 16-minute mark, walked around for a minute, and then sat back down. Impedance continues to decrease as the user sweats and the electrode interface settles.

d. Raw Drowsiness Data

Figure S5 showcases a whole trial's worth of ExG data in both time and frequency domain. No filters were applied to the time-domain representation while the time-frequency spectrogram was generated using a 3000 point FFT using a Hamming window with 2500 sample overlap. Frequency bins above 50 Hz were discarded for visual clarity. The time domain plot exhibits a long settling time most likely due to the large capacitance of the dry electrodes. The spectrogram showcases clear modulation in the alpha band as well as broad spectrum artifacts (most likely caused by motion). There is significant lower frequency (<5 Hz) activity, this is most likely caused by ECG, EOG, and EEG (delta and theta band).

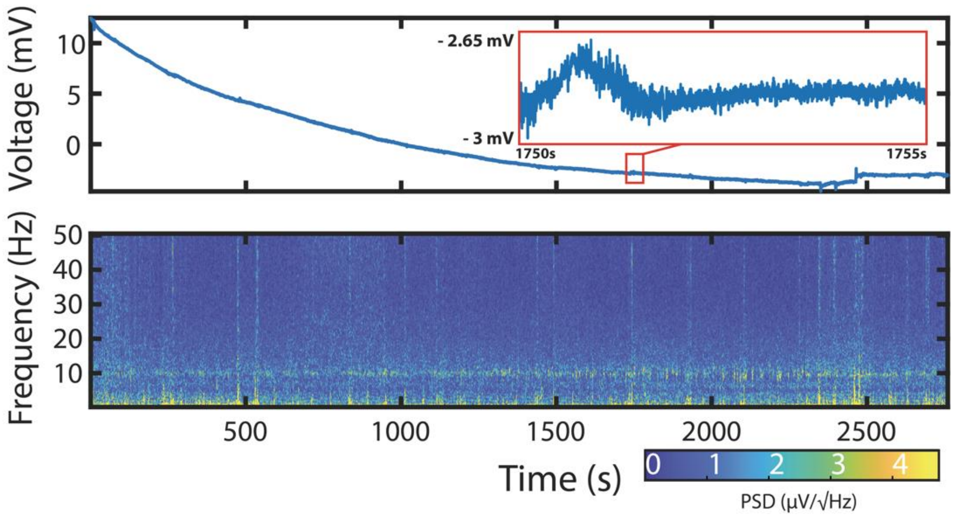

Figure S5: Raw ExG data in time-domain and time-frequency spectrogram from a single drowsiness trial.

e. Drowsiness Detection Model Comparison

The three models presented: logistic regression, support vector machine, and random forest, represent a few commonly used, interpretable machine learning algorithms popular for EEG brain-state classification. All three models performed similarly (Figure 6b – 6g) which is likely due to the simplicity of the models and the stereotypical EEG features for drowsiness. This is further confirmed by observing which individual models performed best for specific subjects with leave-one-user-out validation (Supplemental Table 1). There is no clear, experimental advantage of one model over the other.

Supplemental Table 1: Highest accuracy model for each subject in a leave-one-user-out training regime.

Subject	# Sessions	Classifier Type	Accuracy	Sensitivity	Specificity
**1**	5	LR	96.2	100	95.8
**2**	5	RF	94.9	90.5	97.0
**3**	5	SVM	92	81.0	95.3
**4**	3	RF	91.7	100	90.5
**5**	2	LR	100	100	100
**6**	2	LR	96	100	94.6
**7**	5	SVM	95.1	100	94.3
**8**	4	LR	86.2	100	83.3
**9**	3	SVM	95.2	100	94.4

f. Cross-Validation Accuracy versus number of trials

To observe the effect of larger subject specific training sets, we trained classifiers on an increasing number of trials for the 4 subjects with the most data (5 trials each). Figure S6 demonstrates the resulting model accuracies, sensitivities, and specificities. Across all models, cross-validation accuracy continues to improve as the trials in the training set increase.

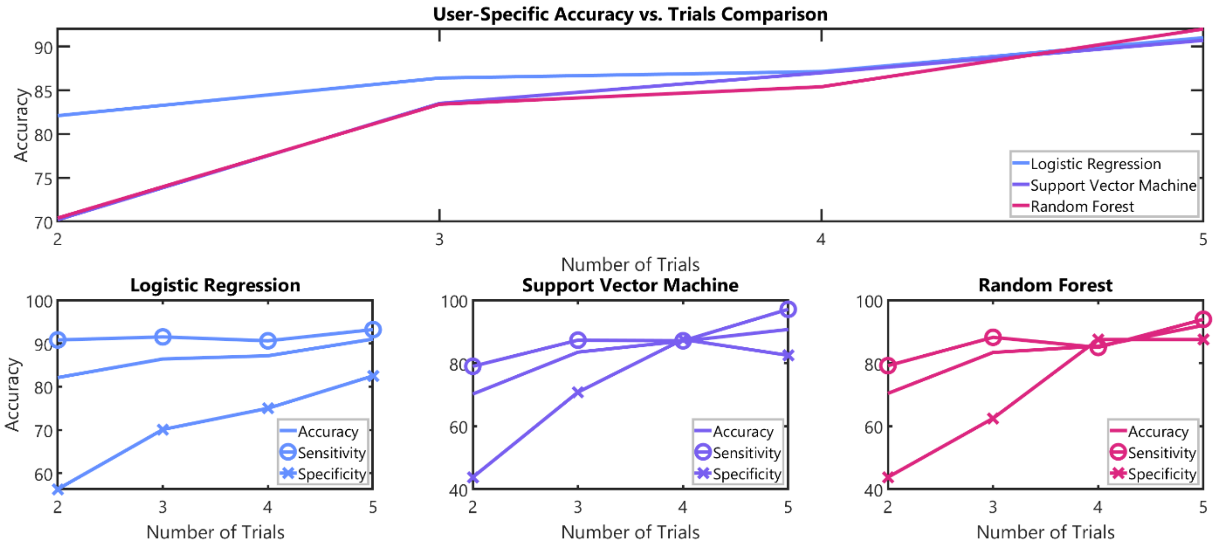

Figure S6: User-specific cross-validation accuracy as the number of trials per user increase.

g. Cross-Validation Accuracy versus number of subjects

To explore the minimum number of subjects required before cross-validation performance begins to saturate, the classifiers were trained with an increasing number of subjects in the training set (use a leave-one-user-out training/cross-validation approach). To control for any individual subject's performance, each classification task was performed on datasets selected by choosing m out of nine subjects. In other words, the two-user cross-validation accuracy was generated by training classifiers on data from (9 choose 2) users (to ensure every possible duo was used to train/validate a model). The final cross-validation results (Fig. S7) from every model would be averaged to get a final accuracy, sensitivity, and specificity. Classifier performance increases as the number of subjects increases. It seems that performance tends to saturate once a dataset consists of six or more subjects (at least five in the testing set and one in the validation set).

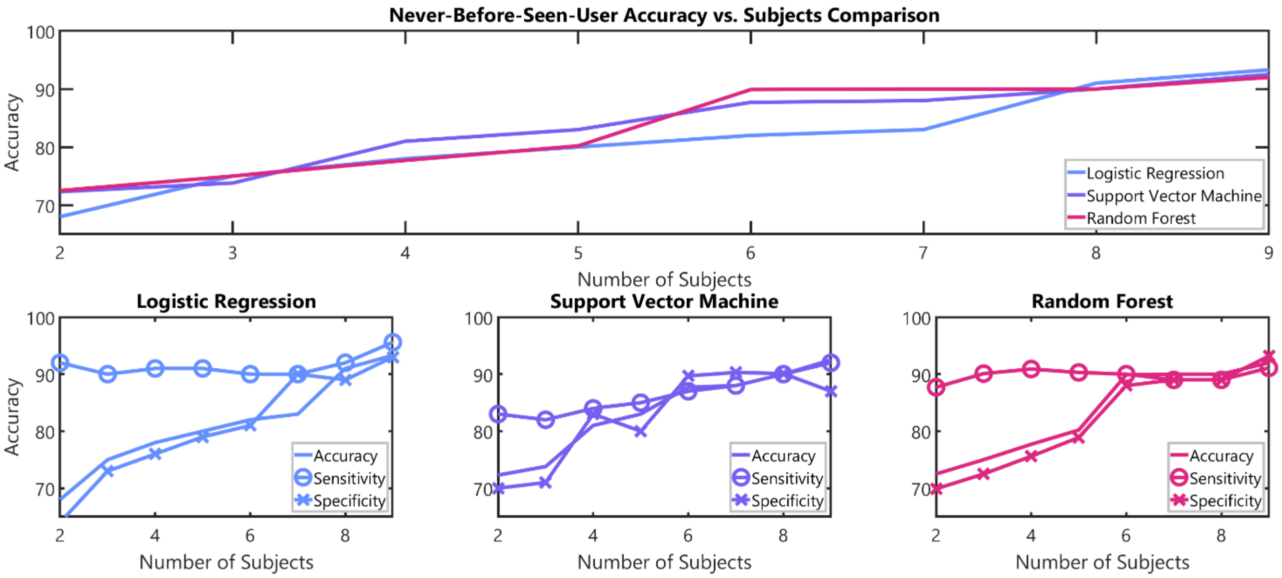

Figure S7: User-specific cross-validation accuracy as the number of subjects in the training set is increased. Accuracy, sensitivity, and specificity all tend to increase as there are more subjects in the training set.

II. Supplemental Methods

a. Plating recipes

The electrode plating procedure requires four solutions: a catalyst, copper plating solution, nickel plating solution, and gold plating solution. The catalyst and copper plating solutions were made in house and the solution recipes can be found in Supplemental Table 2. The catalyst solution was prepared between 60–70 °C and was stirred for approximately 1 hour after all components were added. This solution was prepared in full and can last up to 3 months before the salts precipitate out of solution. The precipitation process proceeds slowly and can be further delayed by periodic stirring between 60-70 °C.

The two main components for an electroless plating solution were a metallic salt and a reducing agent, in this case, copper(II) sulfate and formaldehyde respectively. At a sufficiently high pH, ~12.8, (the solution's pH can be adjusted by adding NaOH), formaldehyde reacts with hydroxide ions in solution to reduce copper ions:

Eq 1: $$Cu^{2+} + 2HCHO + 4OH^{-} \rightarrow Cu + H_2 + 2H_2O + 2HCO_2^{-}$$

In the solution, EDTA was added, which acts as a complexing agent (as copper salts were insoluble at pH > 4), and ferrocyanide, which acts to stabilize the solution over time. To extend the plating solutions lifetime, it was stored without the addition of formaldehyde. At the time of plating, the appropriate quantity of formaldehyde was added to begin the process. At ambient temperatures, the plating process is relatively slow as layers are built at a rate of about <1 µm h^{-1}. As a result, samples were generally submerged in solution overnight in a covered beaker to develop a robust copper layer. A lightly bubbling nitrogen line was added to the solution to ensure even plating, provide light agitation, displace the hydrogen gas product, and limit copper oxidization.

Nickel and gold layers were grown using commercially available solutions from Sigma Aldrich. The nickel-plating process was relatively quick. Copper plated samples would be placed in a 95 – 100 °C bath of nickel-plating solution for 1 – 2 minutes or until visibly coated with nickel. This process is driven by two simultaneous reactions:

Eq 2: $$H_2PO^{2-} + H_2O \rightarrow H_2OPO^{3-} + H_2$$
$$Ni^{++} + H_2 \rightarrow Ni + 2H+$$

These reduction-oxidation reactions occur between nickelous and hypophosphate ions and result in a solid nickel surface and hydrogen gas. The evolved hydrogen not only prevents oxidation but will also agitate the samples. This ensures even coating of highly conductive nickel across the entire sample. The gold layer was applied by heating the gold plating solution (containing cyanide) to about 90 °C and submerging the samples for about 10 min. This process was self-limiting, as gold layers adhere to the copper only and not upon itself. Lastly, this plating solution results in free-floating cyanide in solution and near boiling water. As a result, all plating solutions were used in a fume hood with proper PPE. All solutions can be found in Supplemental Table 2.

Supplemental Table 2: Plating solution components and purpose. All materials were purchased from Sigma-Aldrich.

Solution	Components	Purpose
Catalyst	• 1 L deionized water<br>• 60 mL HCl<br>• 0.25g $PdCl_2$ 12g $SnCl_2$, after $PdCl_2$ completely dissolves	Provides very thin initial palladium layer for copper adhesion
Electroless Copper	• 1000 mL deionized water<br>• 18g $CuSO_4\cdot5H_20$<br>• 48g EDTA<br>• 57.2mg $K_4Fe(CN)_6\cdot3H_20$<br>• 1 mL HCl<br>• NaOH as needed to adjust pH to 12.8<br>• Formaldehyde, when ready for use in 22.5:1 ratio of plating solution:formaldehyde	Plates a thick layer of highly conductive material
Electroless Nickel	Electroless nickel plating solution (Sigma-Aldrich Part Number: 901655)	Prevents grain boundary diffusion between the copper and gold
Electroless Gold	Bright electroless gold plating solution (Sigma-Aldrich Part Number: 901670)	Prevents copper oxidation and improves biocompatibility

b. Microscopy and profilometry

Surface roughness was assessed with light microscopy and stylus profilometry performed on a flat, slide-like sample printed with the same methacrylic polymer. Samples were printed, masked with tape, and subjected to the same plating process described for the electrodes to form selectively patterned films. Microscopy images were taken of each sample using a Nikon Eclipse 50i microscope under 20x magnification, while stylus profilometry measurements were taken with a Dektak stylus profilometer. Surface roughness was calculated by taking the standard deviation of the profilometry measurements.

c. Sheet resistance measurements

Sheet resistance was measured using a Kiethley model 2450 source meter configured for four-point collinear probe measurements (to eliminate the effect of contact resistance). Four-point probe measurements were performed on flat, slide-like sample printed with the same methacrylic polymer and plated with the same electroless plating process as the electrodes.

d. Constant phase element electrode model and fitting

All ESI data was evaluated and fit to a constant phase element (CPE) based model using the Zfit Matlab package. A CPE is an equivalent electrical model for an imperfect double layer and is often used as measure of the electrode-skin interface's non-faradaic impedance. It can be modeled by

Eq 3: $$Z_{CPE} = \frac{1}{(j\omega)^n Q}$$

Where $0 < n \leq 1$. Q is a measure of the magnitude of Z_{CPE} while n fits the bilayer phase offset. The CPE electrode model's impedance can be described by

Eq 4: $$Z_{e,CPE} = R_s + \frac{R_{ct}}{1 + (j\omega)^n Q R_{ct}}$$

Where R_s is spread resistance and R_{ct} is the charge-transfer resistance.

The impedance measurements were modelled using a log normal distribution, *X*, with a standard variable, *V*, mean value, μ, and standard deviation, σ:

Eq 5: $$X = 10^{\mu + \sigma V}$$

e. Alpha modulation experiment and modulation ratio

To monitor alpha modulation with Ear EEG, the subject sat comfortably in a quiet, dark room and were prompted to switch between two states every 30 s over the course of 2 min: an eyes open/focused state and an eyes closed/relaxed state. Mean alpha modulation R_{AM} is defined as the ratio of mean alpha power from eyes closed to eyes open (equation 6).

Eq 6: $$R_{AM} = \frac{P_{avg}(Alpha\ Band_{EyesClosed})}{P_{avg}(Alpha\ Band_{EyesOpen})}$$

f. Drowsiness headset fabrication

Earpieces and electronics were attached to a one-size-fits all athletic sweatband. The WANDmini and accompanying lithium polymer (Li-Po) battery were placed in a custom, 3D printed housing that was sewn into the headband. All electrode and earpiece wires were routed through the headband to minimize any motion artifacts. The selected Li-Po batteries were removable and rechargeable.

g. Earpiece interconnects and wiring

Electrodes were connected to the recording electronics through conventional 30 AWG solid core, copper wire. Due to the quality of the copper, nickel, and gold plating, each electrode is hot soldered to a wire in a location that will not contact the subject's skin (Fig. S8). For added mechanical stability and insulation, all solder joints were covered with UV cured epoxy. Furthermore, electrode wires were bundled with adjacent wires and placed in heat shrink tubing. Each wire was roughly 7 inches such that it could reach a subject's forehead or the corresponding point on the sagittal plane (rear of the

head). Each 30 AWG wire was then soldered to a commercially available .1” jumper cable header so that it can be connected to the WANDmini’s electrode inputs (female .1” headers).

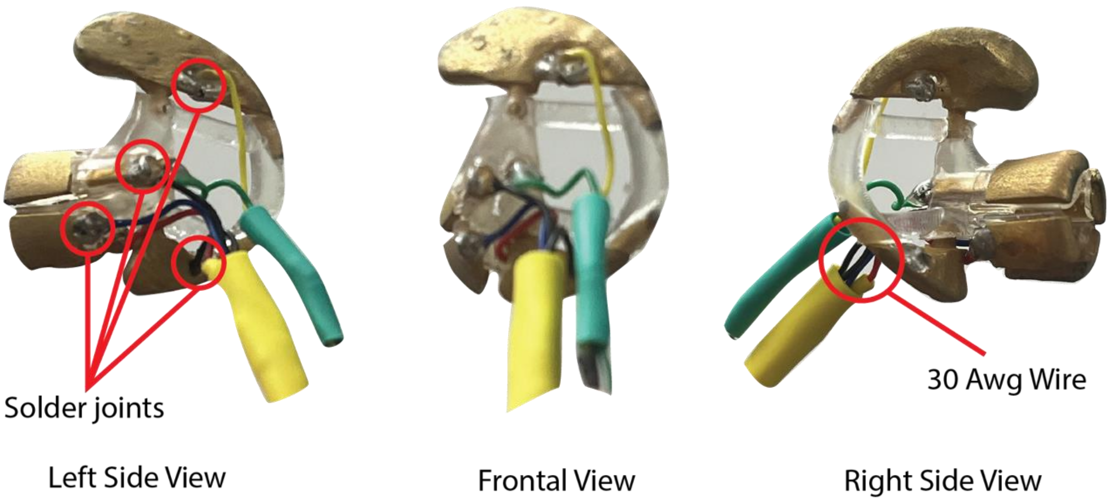

Figure S8: Earpiece close ups showcasing multiple different perspectives and mechanical feature highlights.

h. System Resolution and Noise Floor Calculations

This system was assembled to achieve a high degree of channel flexibility, low power dissipation, and most importantly, meet the required dynamic range of a dry electrode system. The dry electrode earpieces contribute an average ~2 μVrms over 500 Hz (500 Hz being the bandwidth of interest for ECG, EMG, EEG, and EOG). If the maximum input signal is assumed to be 100 mV peak to peak, our required dynamic range can be calculated with the following equation:

$$Dynamic\ Range = 10\log(\frac{Power_{maximum\ signal}}{Power_{minumum\ signal}})$$

Assuming a sinusoidal input, the power of our maximum signal is given by where our amplitude is half of the peak-to-peak voltage:

$$Power_{sinusoid} = (\frac{V_{amp}^2}{2})$$

Then assuming our electrode thermal noise dictates our minimum signal power, the power of our minimum signal is given by:

$$P_{minumum\ signal} = 2\mu V_{rms}^2$$

The resulting dynamic range (84.95 dB) can then be used to calculate the required number of bits to effectively digitize signals (effective number of bits – ENOB) across our input range using the following:

$$ENOB = \frac{Dynamic\ Range - 1.76}{6.02} = 13.82\ bits$$

1.76 is defined as the quantization error of an ideal ADC while 6.02 is the conversion factor between decibels (log10) to bits (log2). WANDmini, with an input range of 100 mV, 1.6 μVrms noise floor, and 15 bit resolution is sufficient for the required dynamic range.

i. Experimental Recording Setup

Subjects sat comfortably in front of the base station radio and host laptop while wearing the WANDmini headband and ear EEG earpieces (Fig. S9). Earpieces sizes were selected per subject based off comfort and electrode-skin impedance. The most comfortable earpieces with the lowest average impedance would be used for the rest of the trial. The headband straps were also tightened to a comfortable point where WANDmini would not move if the user moved their head.

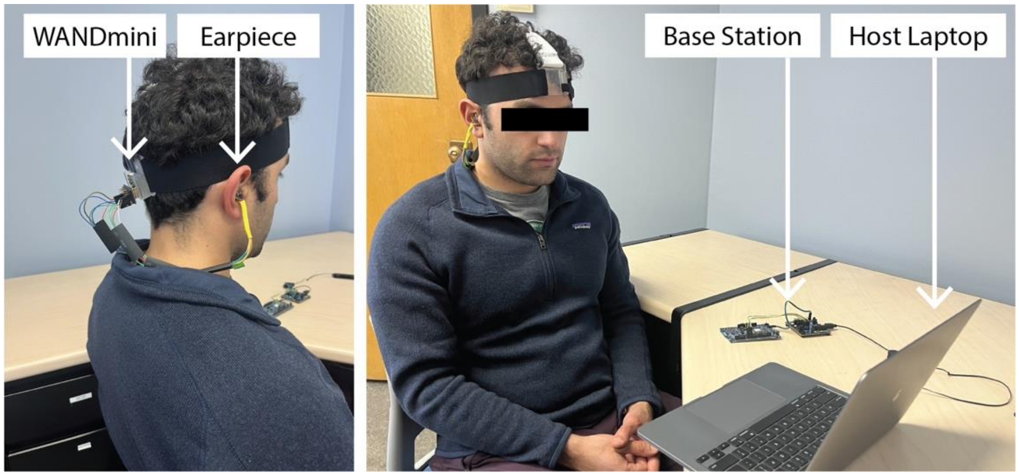

Figure S9: Experimental setup with WANDmini, the earpieces, base station radio, and laptop.

j. Custom Graphical User Interface

The custom graphical user interface (GUI) was developed in Python 3.8 in PyQT and run on the base station during the drowsiness studies. This GUI received and logged the streamed data, plotted all received data for the trial hosts, and provided subjects with the reaction time game and Likert item queries. To prevent users from preparing and “pre-clicking” ahead of time, the reaction time game prompted subjects to enter a specific randomly generated number between 0 and 9. Cues were provided every 60 seconds to minimize interruptions while maximizing drowsiness label granularity. All recorded neural data was saved in a MATLAB array format while the reaction times and Likert items were saved in a CSV format.

k. Event-based metrics

Event-based metrics are used to score drowsiness detection performance. A drowsy event is detected if three minutes of subsequent drowsy epochs are outputted, which minimizes detection latency and false alarms due to noisy classification outputs [3], [4], [5], [6]. Sensitivity represents the true positive rate or the number of correctly classified drowsy events. Similarly, specificity represents the true negative rate or the number of correctly classified alert events. Accuracy is determined by the total number of correctly classified events. Since there is an unequal number of alert and drowsy epochs in this data set, each of these performance metrics is significant to understanding the effectiveness of the drowsiness detection system [7],[16], [50].

Eq 7:
$$Sensitivity = \frac{True\ Drowsy}{True\ Drwosy + False\ Alert}$$

Eq 8:
$$Specificity = \frac{True\ Alert}{True\ Alert + False\ Drowsy}$$

Eq 9:
$$Accuracy = \frac{True\ Drowsy + True\ Alert}{All\ Events}$$

l. Data partitioning across different training and cross-validation schemes

To understand data set requirements with our minimal drowsiness data, three different training and cross-validation regimes were devised. Each scheme had its own partitioning schemes (Fig. S10). User-specific cross validation trained models on n-1 trials for the subject, tested on their remaining trial, and averaged the results after n independent iterations to determine drowsiness detection accuracy for a single subject. Leave-one-trial-out cross validation trained models on 33 of the recorded trails, tested on the remaining trial, and averaged results after all 34 independent iterations to determine the study's overall drowsiness detection accuracy. Leave-one-user-out cross validation trained on recordings from 8 subjects, tested on the remaining subject's recordings, and averaged results after all 9 independent iterations. This evaluated detection accuracy when using population-training and deploying on a never-before-seen subject. In practice, this means that 'leave-one-trial-out' and 'leave-one-user-out' trained models had access to significantly more training data than the user-specific case.

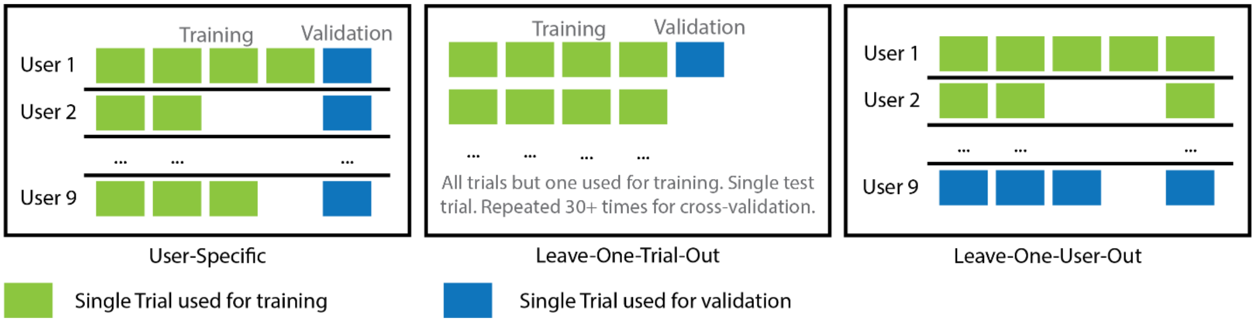

Figure S10: Data partitioning between training and validation sets for different cross-validation schemes.

Supplementary References

[1] R. Kaveh *et al.*, "Wireless User-Generic Ear EEG," *IEEE Trans Biomed Circuits Syst*, vol. 14, no. 4, pp. 727–737, Aug. 2020, doi: 10.1109/TBCAS.2020.3001265.

[2] W. J. Staab, W. Sjursen, D. Preves, and T. Squeglia, "A one-size disposable hearing aid is introduced," *The Hearing Journal*, vol. 53, no. 4. p. 36, 2000. doi: 10.1097/00025572-200004000-00004.

[3] A. Chua, M. I. Jordan, and R. Muller, "SOUL: An Energy-Efficient Unsupervised Online Learning Seizure Detection Classifier," *IEEE J Solid-State Circuits*, vol. 57, no. 8, pp. 2532–2544, Aug. 2022, doi: 10.1109/JSSC.2022.3172231.

[4] Y. Tian and J. Cao, "Fatigue driving detection based on electrooculography: a review," *Eurasip Journal on Image and Video Processing*, vol. 2021, no. 1. Springer Science and Business Media Deutschland GmbH, Dec. 01, 2021. doi: 10.1186/s13640-021-00575-1.

[5] S. Aggarwal and N. Chugh, "Review of Machine Learning Techniques for EEG Based Brain Computer Interface," *Archives of Computational Methods in Engineering*, vol. 29, no. 5. Springer Science and Business Media B.V., pp. 3001–3020, Aug. 01, 2022. doi: 10.1007/s11831-021-09684-6.

[6] N. R. Adão Martins, S. Annaheim, C. M. Spengler, and R. M. Rossi, "Fatigue Monitoring Through Wearables: A State-of-the-Art Review," *Frontiers in Physiology*, vol. 12. Frontiers Media S.A., Dec. 15, 2021. doi: 10.3389/fphys.2021.790292.
