# Supplementary material for: Wireless ear EEG to monitor drowsiness
Source: Nat Commun. 2024 Aug 2;15:6520. doi: 10.1038/s41467-024-48682-7 (PMC11297174; doi:10.1038/s41467-024-48682-7)
Supplement: Supplementary file 3 — Reporting summary [file 41467_2024_48682_MOESM3_ESM.pdf]

nature portfolio

Corresponding author(s): Rikky Muller

Last updated by author(s): Apr 18, 2024

Reporting Summary

Nature Portfolio wishes to improve the reproducibility of the work that we publish. This form provides structure for consistency and transparency in reporting. For further information on Nature Portfolio policies, see our Editorial Policies and the Editorial Policy Checklist.

Statistics

For all statistical analyses, confirm that the following items are present in the figure legend, table legend, main text, or Methods section.

n/a	Confirmed	
☐	☒	The exact sample size (*n*) for each experimental group/condition, given as a discrete number and unit of measurement
☐	☒	A statement on whether measurements were taken from distinct samples or whether the same sample was measured repeatedly
☒	☐	The statistical test(s) used AND whether they are one- or two-sided<br>*Only common tests should be described solely by name; describe more complex techniques in the Methods section.*
☐	☒	A description of all covariates tested
☐	☒	A description of any assumptions or corrections, such as tests of normality and adjustment for multiple comparisons
☐	☒	A full description of the statistical parameters including central tendency (e.g. means) or other basic estimates (e.g. regression coefficient) AND variation (e.g. standard deviation) or associated estimates of uncertainty (e.g. confidence intervals)
☒	☐	For null hypothesis testing, the test statistic (e.g. *F*, *t*, *r*) with confidence intervals, effect sizes, degrees of freedom and *P* value noted<br>*Give P values as exact values whenever suitable.*
☒	☐	For Bayesian analysis, information on the choice of priors and Markov chain Monte Carlo settings
☒	☐	For hierarchical and complex designs, identification of the appropriate level for tests and full reporting of outcomes
☒	☐	Estimates of effect sizes (e.g. Cohen's *d*, Pearson's *r*), indicating how they were calculated

Our web collection on statistics for biologists contains articles on many of the points above.

Software and code

Policy information about availability of computer code

Data collection	All data collection was performed using embedded C, Verilog, and Python 3.5. Certain verilog and embedded C drivers are proprietary to the FPGA/microcontroller manufacturer, but all python packages used were open-source (numpy, scipy, etc.).
Data analysis	All analysis was performed with Python 3.5 with the following packages: math, h5py, numpy, pandas, scipy, matplotlib, itertoosl, sklearn, pympler, multiprocessing, and joblib.

For manuscripts utilizing custom algorithms or software that are central to the research but not yet described in published literature, software must be made available to editors and reviewers. We strongly encourage code deposition in a community repository (e.g. GitHub). See the Nature Portfolio guidelines for submitting code & software for further information.

Data

Policy information about availability of data

All manuscripts must include a data availability statement. This statement should provide the following information, where applicable:

- Accession codes, unique identifiers, or web links for publicly available datasets
- A description of any restrictions on data availability
- For clinical datasets or third party data, please ensure that the statement adheres to our policy

Pertinent python code (for classification) is available on github. While the recorded data is not clinically available, our human subject study protocol does only allows

for the public release of any recorded biological, neural, or physiological data. This will be done by adding verified users to a private github repository with instructions in the manuscript. We are beginning the process of updating our IRB so that the data can be publicly available without any hurdles.

Research involving human participants, their data, or biological material

Policy information about studies with human participants or human data. See also policy information about sex, gender (identity/presentation), and sexual orientation and race, ethnicity and racism.

Reporting on sex and gender	Due to the small size of the dataset, the authors do not have enough information to determine if sex or gender are pertinent to the published results. Sex was self-reported by subjects (7 males, 2 females) for use in future studies that will analyze potential neurological biomarker differences that would be relevant to brain-state classification. Consent was obtained to release anonymized metadata about the study protocol.
Reporting on race, ethnicity, or other socially relevant groupings	No race, ethnicity, or any other social grouping information was collected or used in the study procedure.
Population characteristics	All subjects were between the ages of 18 and 27 and had no history of sleep disorders.
Recruitment	Participants were recruited through a volunteer form emailed to various University departments. No compensation was provided to participants.<br><br>Due to the potential participant pool, there is certainly potential for selection bias (primarily age related). Research results may be impacted as the dataset grows to include a wider age range and a more even sex split.
Ethics oversight	The user study, subject recruitment, and all data analysis was approved by UC Berkeley's Institutional Review Board (CPHS protocol ID: 2018-09-11395).

Note that full information on the approval of the study protocol must also be provided in the manuscript.

Field-specific reporting

Please select the one below that is the best fit for your research. If you are not sure, read the appropriate sections before making your selection.

☒ Life sciences ☐ Behavioural & social sciences ☐ Ecological, evolutionary & environmental sciences

For a reference copy of the document with all sections, see nature.com/documents/nr-reporting-summary-flat.pdf

Life sciences study design

All studies must disclose on these points even when the disclosure is negative.

Sample size	9 subjects
Data exclusions	None. All recorded data was saved for use in the study.
Replication	EEG collection can be replicated with appropriate hardware. Software results can be replicated with the provided data.
Randomization	To the author's best knowledge, this is non applicable.
Blinding	All identifiable information such as name, age, and gender have been destroyed. Data is completely blind

Reporting for specific materials, systems and methods

We require information from authors about some types of materials, experimental systems and methods used in many studies. Here, indicate whether each material, system or method listed is relevant to your study. If you are not sure if a list item applies to your research, read the appropriate section before selecting a response.

Materials & experimental systems

n/a	Involved in the study
☒	☐ Antibodies
☒	☐ Eukaryotic cell lines
☒	☐ Palaeontology and archaeology
☒	☐ Animals and other organisms
☒	☐ Clinical data
☒	☐ Dual use research of concern
☒	☐ Plants

Methods

n/a	Involved in the study
☒	☐ ChIP-seq
☒	☐ Flow cytometry
☒	☐ MRI-based neuroimaging

Plants

Seed stocks	N/A
Novel plant genotypes	N/A
Authentication	N/A
